# Social Support and Coping Style of Medical Residents in China: The Mediating Role of Psychological Resilience

**DOI:** 10.3389/fpsyt.2022.888024

**Published:** 2022-06-20

**Authors:** Chao Xu, Yongyi Wang, Zongqin Wang, Biao Li, Chuandong Yan, Sheng Zhang, Bei Chen, Di Zhang, Juan Peng

**Affiliations:** ^1^Department of Psychiatry, Wuhan Mental Health Center, Wuhan, China; ^2^Department of Senile Psychosomatic, Wuhan Hospital for Psychotherapy, Wuhan, China; ^3^Tongji Medical College, Huazhong University of Science and Technology, Wuhan, China

**Keywords:** residents, psychological resilience, social support, coping style, mediation

## Abstract

**Objectives:**

Recent surveys have paid insufficient attention to the psychological status of medical residents, but medical residents, as a special group of medical workers, need to be focused on. This study aimed to investigate medical residents' levels of social support, psychological resilience, and coping style, and explore the mediating role of psychological resilience, which can ultimately provide a new theoretical basis for improving medical residents' psychological status and quality of work and life.

**Methods:**

A total of 577 medical residents from China were investigated by an online questionnaire, using convenience sampling. Associations between social support, psychological resilience, and coping styles were assessed using Pearson correlation analysis. The mediating effect of psychological resilience was tested using SPSS Process.

**Results:**

Positive correlations between social support, psychological resilience and coping style were found (*r* = 0.474, *P* < 0.001; *r* = 0.473, *P* < 0.001; *r* = 0.353, *P* < 0.001). The mediating effect of psychological resilience in the relationship between social support and coping style was significant (95% CI: 0.168, 0.384), and accounted for 25.84%.

**Conclusion:**

Attention should be paid to the psychological status of medical residents, and social support and psychological flexibility can be used to increase the enthusiasm for their coping style and promote their mental health.

## Introduction

Medical residents are primary care physicians in hospitals, mainly responsible for the basic clinical medical work, including receiving patients, recording the course of the disease, and performing certain clinical operations, but need to receive the guidance and supervision of superior doctors (e.g., attending doctors and above). Medical residents must develop specific skills in their chosen area during their residency to maintain the quality of patient care. However, during this period, they suffer from various problems, such as lack of sleep, heavy workload, and salary dissatisfaction (1). A review study showed that long working hours, as well as their negative impact on personal life, are the most common causes of personnel loss in the department of general surgery (2). Studies have focused on the mental health problems of medical residents and speculated that it may have direct or long-term serious consequences for patients or doctors themselves, but existing research has focused more on occupational burnout (2, 3). The main conclusion was that occupational burnout in medical residents was more severe and higher than that in attendings (2, 4). Occupational burnout was thought to be associated with poor psychological status (3), but few studies have reported the psychological status of medical residents.

Actually, psychological stress should be paid more attention to in the current environment of the coronavirus disease pandemic. The task of preventing and controlling infectious diseases is aggravated, medical human resources are scarce, the pressure on medical staff is higher, and the psychological burden is heavier. Several studies have proposed a potential impact of the pandemic on healthcare professionals' mental health (5–7). It has emphasized that simultaneous mental health informed interventions are necessary to promote coping (7).

Under long-term great pressure, psychological resilience has received some attention as one of the characteristics reflecting psychological status (8, 9). Psychological resilience is the ability to maintain the persistence of one's orientation toward existential purposes (10). It constitutes a horizontal attitude that can be understood as the ability to perseverance overcome difficulties experienced in different areas of one's life and a good awareness of oneself and one's internal coherence by activating a personal growth project (10).

Also, there is evidence that psychological resilience may be associated with coping style and social support (11–13). Coping styles refer to the cognitive and behavioral changes that result from the management of an individual's specific external/internal stressors (12). Social support refers to various types of assistance from social networks and can be formal and/or informal, including emotional and physical support (13). In a cross-sectional study, psychological resilience was a significant factor influencing positive coping styles among Chinese undergraduates (12). Psychological resilience can also affect the way diabetic patients solve problems (14). This may be because individuals with high psychological resilience are more likely to show positive cognitive and coping styles in the face of stressful events; conversely, individuals with low psychological resilience are more negative and prone to negative emotions. Alternatively, other evidence suggested that social support provided by families and medical health care personnel can significantly increase psychological resilience in patients undergoing colorectal cancer surgery (15). And social support was an important factor in the development of psychological resilience among elderly caregivers in Singapore and groups of breast cancer patients in China (13, 16). Perhaps this is because individuals with high levels of psychological resilience will take the initiative to use the source of support around them to solve the problems encountered. It is worth noting that social support, a psychological and material resource provided by a social network (17), is a strong backing for individuals when faced with stress and difficulties, helping to enhance confidence in coping with frustration and affecting the choice of coping style for individuals. Studies have shown that social support could reduce stress, reduce the impact of stressful situations, prepare individuals for difficult conditions, and enhance their coping ability (18).

The aim of this study was (1) to verify the relationship between medical residents' psychological resilience, social support, and coping style, (2) and to explore what role psychological resilience plays between the two, (3) ultimately provide a new theoretical and practical basis for improving medical residents' psychological status and quality of work and life.

Therefore, the following two hypotheses can be proposed:

**Hypothesis 1**: Psychological resilience, coping style, and social support of medical residents are associated with each other.
**Hypothesis 2**: Medical residents' psychological resilience plays a mediating role between social support and coping styles.

## Materials and Methods

### Participants and Data Collection

Medical residents were selected using non-probability sampling among the four largest hospitals in Hubei Province. These four hospitals are the largest, highest ranked, with the highest number of beds and outpatient visits in Hubei Province, and also have many relatively standardized medical teams. The questionnaire was released through the online platform and was volunteered by medical residents to participate in the survey. The questionnaire was collected for 2 weeks. To ensure the quality of the questionnaire, questionnaires with an answering time of fewer than 3 mins were excluded, and each IP could only fill in it once, and a total of 577 questionnaires were finally collected.

### Tools

#### Coping Style

The Chinese Trait Coping Style Questionnaire (TCSQ) was used to measure medical residents' coping styles, which included two dimensions: positive and negative coping. Each dimension consists of 10 items on a 5-point Likert-type scale, ranging from 1 (absolutely no) to 5 (absolutely yes), respectively. The difference was obtained by subtracting the total negative coping style score from the total positive coping style score. When the difference is positive, it indicates that individuals prefer a positive coping style. The validity and reliability of the TCSQ have been verified (17, 19).

#### Social Support

Social support was measured using the Social Support Rating Scale (SSRS) with 10 items (20). The total SSRS score ranges from 12 to 66 points, and higher scores on this measure indicate a higher level of social support. The validity and reliability of this scale in the Chinese population have been validated (21).

#### Psychological Resilience

The Connor-Davidson Resilience Scale (CD-RISC) was chosen for this study to measure the level of psychological resilience in medical residents (22). The scale is a self-rating scale that was currently translated into multiple versions and was widely used for the measurement of psychological resilience in different populations and different situations (23–25). Chinese investigators translated the scale into a Chinese version with internal consistency reliability of 0.91 and Cronbach's α ranging between 0.60 and 0.88 (26). The CD-RISC contains 25 items with responses ranging from 5 points for all items as follows: not true at all (0), rarely true (1), sometimes true (2), usually true (3), and true almost all the time (4). This scale was rated based on how the participant has felt over the past month. The total score ranges from 0 to 100, with a higher score indicating more resilience (22).

#### Sociodemographic

Sociodemographic data included gender (male = 1, female = 2), age, marital status (married = 1, unmarried = 2), whether they were the only child (yes = 1, no = 2) and hometown (Wuhan = 1, outside Wuhan within Hubei province = 2, outside Hubei province = 3).

### Data Analysis

**Figure 1:**
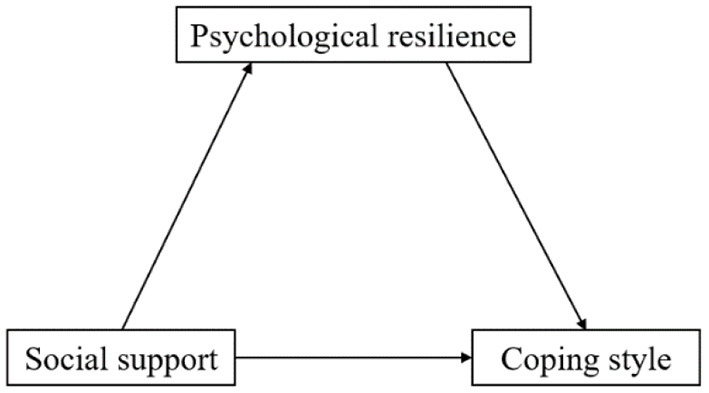
Conceptualized model.

Statistical analysis was performed using SPSS version 24. Categorical variables were presented as frequencies and percentages. Continuous variables were expressed as mean ± standard deviation (SD). Associations between variables were assessed using Pearson correlation analysis. The SPSS Process was used to test the effect of social support on coping style through psychological resilience (Model 4). The significance of the mediation effect was tested by Bootstrap and a 95% confidence interval (CI) for the mediation effect was calculated by sampling 5,000 times in the original data using repeated random sampling. If the 95%CI does not include 0, the path is significant. The conceptualized model was shown in Figure 1.

## Result

Detailed descriptions of sociodemographic characteristics and main variables were given in Table 1. The main participants in this study were unmarried, with a similar proportion of males (49.4%) and females (50.6%). More than half of the participants were only child (53.2%), and most physicians' hometowns were outside Hubei Province (79.9%).

**Table 1:** Sample characteristics and primary variables (*N* = 577).

	***n***	**Percent (%)/Mean ±SD**
**Gender**		
Male	285	49.4
Female	292	50.6
**Age**		22.58 ± 2.944
**Marital status**		
Married	10	1.7
Unmarried	567	98.3
**Only child or not**		
Yes	307	53.2
No	270	46.8
**Homeland**		
Wuhan	65	11.3
Outside Wuhan, Hubei	51	8.8
Outside Hubei	461	79.9
**Coping style**		7.58 ± 11.038
**Social support**		27.60 ± 5.121
**Psychological resilience**		65.08 ± 19.862

The results of correlation analysis (Table 2) showed that only age was positively correlated with coping style (*r* = 0.102, *P* < 0.05). There were positive relationships between social support, psychological resilience and coping style (*r* = 0.474, *P* < 0.001; *r* = 0.473, *P* < 0.001; *r* = 0.353, *P* < 0.001).

**Table 2:** Correlation between sociodemographic data and main variables.

	**1**	**2**	**3**	**4**	**5**	**6**	**7**	**8**
1. Gender	1							
2. Age	0.011	1						
3. Marital status	0.002	−0.444^***^	1					
4. Only child or not	0.072	0.185^***^	−0.062	1				
5. Homeland	−0.054	−0.080	0.057	0.087^*^	1			
6. Coping style	−0.072	0.102^*^	−0.035	−0.037	−0.032	1		
7. Social support	−0.020	0.048	−0.016	0.055	0.026	0.474^***^	1	
8. Psychological resilience	−0.066	0.030	−0.029	−0.024	−0.056	0.473^***^	0.353^***^	1

^*^P < 0.05, ^***^P < 0.001. Significant results between the main variables were shown in bold.

Results of mediation analysis were presented in Table 3. Social support had a significant effect on psychological resilience (β = 1.385, *P* < 0.001) and psychological resilience had a significant effect on coping style (β = 0.192, *P* < 0.001). Total effect (95% CI: 0.871, 1.182), direct effect (95% CI: 0.607, 0.916), and indirect effect of psychological resilience (95% CI: 0.168, 0.384) were all significant. And the indirect effect accounted for 25.84%.

**Table 3:** Total, direct, and indirect effect of social support on coping style via psychological resilience.

	**Coeff./Effect**	***P***	**95%CI**	
			**Lower**	**Upper**
X → M	1.385	<0.001	1.087	1.683
X → Y	0.761	<0.001	0.607	0.916
M → Y	0.192	<0.001	0.152	0.231
Total effect	1.027	<0.001	0.871	1.182
Direct effect	0.761	<0.001	0.607	0.916
Indirect effect	0.265		0.168	0.384
Proportion	25.84%			

*X, social support; M, psychological resilience; Y, coping style*.

## Discussion

The level of psychological resilience of medical residents in the present study was 65.08 ± 19.86, similar to the results of a study of Turkish nurses (64.28 ± 15.99) (27). This study explored the role of psychological resilience in the relationship between social support and coping style in medical residents. And the results showed that social support and psychological resilience were positively correlated with coping style. Psychological resilience played a part of the intermediary role in the relationship between the two. These results support our hypothesis.

Our findings showed that individuals with a high level of social support were more likely to prefer a positive coping style. Social support is widely recognized as a valuable resource, including tangible forms of assistance that individuals receive from family, friends, and others (28). On the one hand, a high level of social support often means that there are more available resources (28); on the other hand, emotional encouragement and understanding derived from family or friends help alleviate emotions (18, 29) and is the hope that people have positive psychological energy (30). During stressful events, including disasters, disasters, and disease outbreaks, social and peer support is an important protective factor for the overall mental health of medical staff themselves (5). Moreover, lack of support and understanding from family members and relatives, and lack of people's recognition of medical work can affect individuals' coping (8); Clinicians have begun to recognize the importance of social support in the form of family and friends for patients (28). The previous study has included more social support from mast cell patients in adaptive coping strategies, implying the impact of social support on coping (31). At the same time, patients' avoidance-type coping activities are associated with a low frequency of seeking social support (31). Social support was also considered to have a direct and buffering effect on patients' health and emotional adaptation (32) and was one of the effective strategies to improve the coping style and quality of life of cancer patients (32).

Social support has also been shown to influence coping style through psychological resilience. Social support can provide favorable external environmental conditions for the development of psychological resilience, for example, old adults who perceived higher support from social networks could prompt them to actively adapt to stressors resulting from migration and aging rather than avoid them (33). And the current study showed that social media use can enhance psychological resilience and increase the level of perceived social support to meet individuals' psychosocial needs in a fast-paced and rapidly changing world (18). Particularly, in crises, social media plays two important roles: it facilitates timely access to information from informal and official sources, and it also links people with their loved ones and the community who provide relief, help, and support (34). Thus, good social support can give people positive energy and support, promote them to view stress events optimistically, attenuate the negative effects caused by stress events, and improve hardiness. This was consistent with the findings of a study of caregivers of psychiatric patients (35). Providing social support to caregivers of patients with schizophrenia would improve their psychological resilience and may also help to improve their coping with these deficits (35). Studies in the nursing field have also shown that social support was a positive factor in the psychological resilience among midwife candidates, and as psychological resilience increased, self-confidence and problem-solving ability were also enhanced (36). In settings where epidemics are rampant nowadays, psychological resilience has still received continuous attention, suggesting its undeniable importance. Some investigators have recommended the development and implementation of strategies aimed at improving the psychological resilience of HCWs through evidence-based education and training to strengthen HCWs' defense against the various mental and psychological consequences of the pandemic (37, 38). Therefore, the focus of this study is of great significance.

This study has several limitations. First, a cross-sectional study was conducted at a single time point, and no conclusion on causality can be drawn. Second, this study collected data in one city, which may have introduced bias and limited the generalizability of the findings. Therefore, future studies with participants from different regions are warranted. Third, this study employed a self-report questionnaire, which may have contributed to reporting bias.

## Conclusion

The study focused on medical residents' social support, psychological resilience, and coping style. The results of the study confirmed that there was a significant positive correlation among the three, and at the same time, a mediating effect of psychological resilience was found. Therefore, we believe that attention to the psychological status of medical residents should be strengthened, and attempts should be made to increase social support and psychological resilience to increase the enthusiasm for coping styles and ultimately improve their mental health status.

## Data Availability Statement

The raw data supporting the conclusions of this article will be made available by the authors, without undue reservation.

## Ethics Statement

The studies involving human participants were reviewed and approved by the Ethics Committee of Wuhan Mental Health Center, Huazhong University of Science and Technology. The patients/participants provided their written informed consent to participate in this study.

## Author Contributions

CX and JP: conceptualization, data curation, formal analysis, methodology, writing—original draft, writing—review and editing, validation, and visualization. YW: formal analysis, methodology, writing—original draft, writing—review and editing, validation, and visualization. ZW and BL: data curation, formal analysis, and writing—review and editing. CY, SZ, BC, and DZ: data curation, writing—review and editing, and validation. All authors contributed to the article and approved the submitted version.

## Funding

This work was supported by the Wuhan Municipal Health Commission (WX18Q35).

## Conflict of Interest

The authors declare that the research was conducted in the absence of any commercial or financial relationships that could be construed as a potential conflict of interest.

## Publisher's Note

All claims expressed in this article are solely those of the authors and do not necessarily represent those of their affiliated organizations, or those of the publisher, the editors and the reviewers. Any product that may be evaluated in this article, or claim that may be made by its manufacturer, is not guaranteed or endorsed by the publisher.
